# Domestication and selection footprints in Persian walnuts (*Juglans regia*)

**DOI:** 10.1371/journal.pgen.1010513

**Published:** 2022-12-07

**Authors:** Xiang Luo, Huijuan Zhou, Da Cao, Feng Yan, Pengpeng Chen, Jiangtao Wang, Keith Woeste, Xin Chen, Zhangjun Fei, Hong An, Maria Malvolti, Kai Ma, Chaobin Liu, Aziz Ebrahimi, Chengkui Qiao, Hang Ye, Mengdi Li, Zhenhua Lu, Jiabao Xu, Shangying Cao, Peng Zhao

**Affiliations:** 1 College of Agriculture, Henan University, Kaifeng, Henan, China; 2 Key Laboratory of Resource Biology and Biotechnology in Western China, Ministry of Education, College of Life Sciences, Northwest University, Xi’an, Shaanxi, China; 3 Zhengzhou Fruit Research Institute, Chinese Academy of Agricultural Sciences, Zhengzhou, China; 4 Xi’an Botanical Garden of Shaanxi Province, Xi’an, China; 5 College of Forestry, Northwest A&F University, Yangling, Shaanxi, China; 6 Laboratory of Functional Plant Biology, Department of Biology, Ghent University, Ghent, Belgium; 7 USDA Forest Service Hardwood Tree Improvement and Regeneration Center (HTIRC), Department of Forestry and Natural Resources, Purdue University, West Lafayette, Indiana, United States of America; 8 Shandong Institute of Pomology, National Germplasm Repository of Walnut and Chestnut, Tai’an, China; 9 Boyce Thompson Institute for Plant Research, US Department of Agriculture (USDA) Robert W. Holley Center for Agriculture and Health, Cornell University, Ithaca, New York, United States of America; 10 Bioinformatics and Analytics Core, University of Missouri, Columbia, Missouri, United States of America; 11 Research Institute on Terrestrial Ecosystems, National Research Council, Porano, Terni, Italy; 12 Xinjiang Academy of Agricultural Sciences, Urumqi, China; 13 BGI Genomics, BGI-Shenzhen, Shenzhen, China; Peking University, CHINA

## Abstract

Walnut (*Juglans*) species are economically important hardwood trees cultivated worldwide for both edible nuts and high-quality wood. Broad-scale assessments of species diversity, evolutionary history, and domestication are needed to improve walnut breeding. In this study, we sequenced 309 walnut accessions from around the world, including 55 *Juglans* relatives, 98 wild Persian walnuts (*J*. *regia*), 70 *J*. *regia* landraces, and 86 *J*. *regia* cultivars. The phylogenetic tree indicated that *J*. *regia* samples (section *Dioscaryon*) were monophyletic within *Juglans*. The core areas of genetic diversity of *J*. *regia* germplasm were southwestern China and southern Asia near the Qinghai-Tibet Plateau and the Himalayas, and the uplift of the Himalayas was speculated to be the main factor leading to the current population dynamics of Persian walnut. The pattern of genomic variation in terms of nucleotide diversity, linkage disequilibrium, single nucleotide polymorphisms, and insertions/deletions revealed the domestication and selection footprints in Persian walnut. Selective sweep analysis, GWAS, and expression analysis further identified two transcription factors, *JrbHLH* and *JrMYB6*, that influence the thickness of the nut diaphragm as loci under selection during domestication. Our results elucidate the domestication and selection footprints in Persian walnuts and provide a valuable resource for the genomics-assisted breeding of this important crop.

## Author summary

Walnut (*Juglan*s) species are economically important hardwood trees. The Persian walnut as a woody nut crop is limited by an incomplete understanding of the diversity and spatial genetic structure of the species’ wild germplasm, and the relationship of the secondary germplasm pool of walnut (wild and landrace walnuts) to improved cultivars. To better understand walnut’s breeding, we sequenced the genomes of 309 walnut accessions from around the world. The results revealed core areas of genetic diversity of Persian walnut germplasm were southwestern China and southern Asia near the Qinghai-Tibet Plateau and the Himalayas. The genome-wide scan for selective sweeps, genome-wide association studies (GWAS), and expression analysis identified *JrbHLH* and *JrMYB6* transcript factors related to the thickness of the diaphragm that were under selection during domestication. Our results elucidate the genetic diversity of walnut and its evolution as a species and a crop. We identified centers of wild genetic diversity that could contribute to breeding and sustainable improvement.

## Introduction

Persian or common walnut (*Juglans regia* L.; Juglandaceae, Fagales) is a large, wind-pollinated, diploid (2n = 32), monoecious, heterodichogamous, long-lived tree. It is grown worldwide [1–3] for its edible nuts and high-quality wood [4–6]. Nearly 500 varieties and cultivars of Persian walnut have been recorded around the world [6–10]. The relationships between the current distribution of genetic diversity, its ancient centres of diversity, and its histories of domestication and spread by humans remain open questions [6–10]. The authors of a recent study suggested that *J*. *regia* originated as an ancient hybrid between American and Asian walnut lineages in the late Pliocene (3.45 million years ago, Mya) [11]. It is generally accepted that after the last glaciation, *J*. *regia* survived and grew in isolated stands in the foothills of the Western Himalayas from the Kashmir region to Tajikistan and Kyrgyzstan [9–13] and in South Asia [9]. Persian walnut was also a part of the ancient Chinese flora [14]. C-dated leaf fossils and carbonized nuts found in Shandong and Hebei provinces were shown to be ca. 7,335 ± 100 years old [12,14]. Some researchers have suggested that the presence of additional centres of diversity of walnuts were in Eurasia and even southern Europe [6,7,10,12,15]. Whatever the distribution of walnut before its human-mediated dispersal, the current species range is the product of long-term and complex interactions between biogeography, climate, and human forces [10,12,16–17]. Indeed, the biogeography of *J*. *regia* is complicated by its long history of human cultivation and its use as a traditional nut crop in many Old World cultures. Because of its ancient association with humans and the movement of walnuts via regional- and even continental-scale trade routes, apparently wild or (possibly) feral populations of walnuts are now found in isolated favourable habitats from China to the mountains of the Iberian Peninsula [9,10,12]. Population genetics and comparative genomics help the footprints of domestication and migration, which have been applied successfully to both annual crops (e.g., rice, soybean, and cotton) [18–21] and perennials (e.g., apple, pear, peach, and chestnut) [22–25].

Programs to produce improved, clonal varieties of Persian walnut began in the mid-20^th^ century, so modern cultivars have short pedigrees, although the selection and dispersal of walnuts by humans have been occurring in Asia and Europe for millennia [7,10,12,16]. Traits considered desirable for modern international markets include a light kernel colour and a tight shell seal. It is still unclear which other traits may have been selected by humans in diverse and dispersed villages across the species’ range. The walnut landraces propagated by local communities as seedlings represent a valuable resource as a secondary germplasm pool for breeders. Theoretically, germplasm pools propagated as seedlings are more genetically diverse than those created with controlled crosses within a pedigree [5,26–27]. Landraces might also contain linkage blocks of genes for adaptations to biotic and abiotic stresses, particularly in the source environment [28–30]. The value of these landraces is compounded by their preselection for traits desired by human consumers and cultivators. The secondary germplasm pool for walnut is not well characterized; although some wild or apparently wild germplasm has been collected [31], most of the resources are still maintained *in situ* in remote locations, and the relationship between landrace samples and already characterized populations is unclear. Wild or landrace walnut populations may be descendants of truly autochthonous local sources, feral trees, descendants of trees dispersed from distant locations by humans, or a combination of these sources. The origins of walnut populations and the effects of human selection during domestication may be revealed by a detailed genetic comparison of wild, landrace, and cultivar populations. The availability of single nucleotide polymorphism (SNP) markers for rapid and highly automated genotyping makes them ideal for genetic diversity studies, genomic selection analysis, and genome-wide association studies (GWAS).

Here, we sequenced 309 diverse walnut accessions from around the world, including 55 *Juglans* relatives, 98 (presumed) wild *J*. *regia* accessions, 70 *J*. *regia* landraces, and 86 *J*. *regia* cultivars. We provide genomic evidence for elucidating the intraspecific genetic relationships of walnut source populations, the historical dynamics of *J*. *regia* cultivation, and candidate loci under selection during Persian walnut domestication.

## Results

### Genetic variation of walnut accessions

To understand the diversity of Persian walnut, we sequenced 309 *Juglans* accessions, including wild walnuts, landrace walnuts, cultivars, and related species (S1 Table). We evaluated a total of 4,643 Gb of high-quality, cleaned sequence data, with an average of 15.03 Gb per accession, which is equivalent to an approximately 29× coverage of the ~ 568 Mb walnut genome [5,26]. These sequences were aligned to the genome of the Persian walnut ‘Chandler’ [5,26], with an average mapping rate of 98.42% (S1 Table). We identified 25,063,036 single nucleotide polymorphisms (SNPs) and 3,845 insertions and deletions (InDels) at the genome level (S1C and S1D Fig).

### Genetic diversity and linkage disequilibrium in the genomes of walnuts

The nucleotide diversity (π) of walnut at the whole-genome level across all walnut accessions was 4.5 × 10^− 3^ (Table 1). The patterns of differences in diversity (π) and SNP and InDel frequencies revealed genetic differentiation between wild walnuts and domesticated walnuts (cultivars and landraces) (S1C, S1D, and S1F Fig). Wild walnut genomes contained notably higher nucleotide diversity (4.56 × 10^− 3^) than landrace walnuts (4.44 × 10^− 3^) and cultivated walnut (4.39 × 10^− 3^) genomes (Table 1). The genome-level comparisons showed that the population differentiation (F index, F_ST_) between wild walnuts and landrace walnuts was similar to that between wild walnuts and cultivars, but there was a relatively low F_ST_ value between landraces and cultivars based on SNPs and InDels, indicating that modern breeding has not substantially influenced the genome of walnuts beyond the initial effects of domestication.

**Table 1:** Nucleotide diversity and linkage disequilibrium (LD) decays in wild walnuts, walnut landraces, and walnut cultivars.

Groups	Number of samples	SNP (N)	SNP/kb	θπ	*Tajima’s* D	r^2^	Half distance (Kb)
Wild walnuts	98	8,387,336	14.39	4.56×10^−3^	2.29	0.2926	3.8
Landraces	70	8,367,894	14.40	4.44×10^−3^	2.30	0.2894	7.6
Cultivars	86	8,307,806	14.47	4.39×10^−3^	2.19	0.2925	7.4

**Fig 1:**
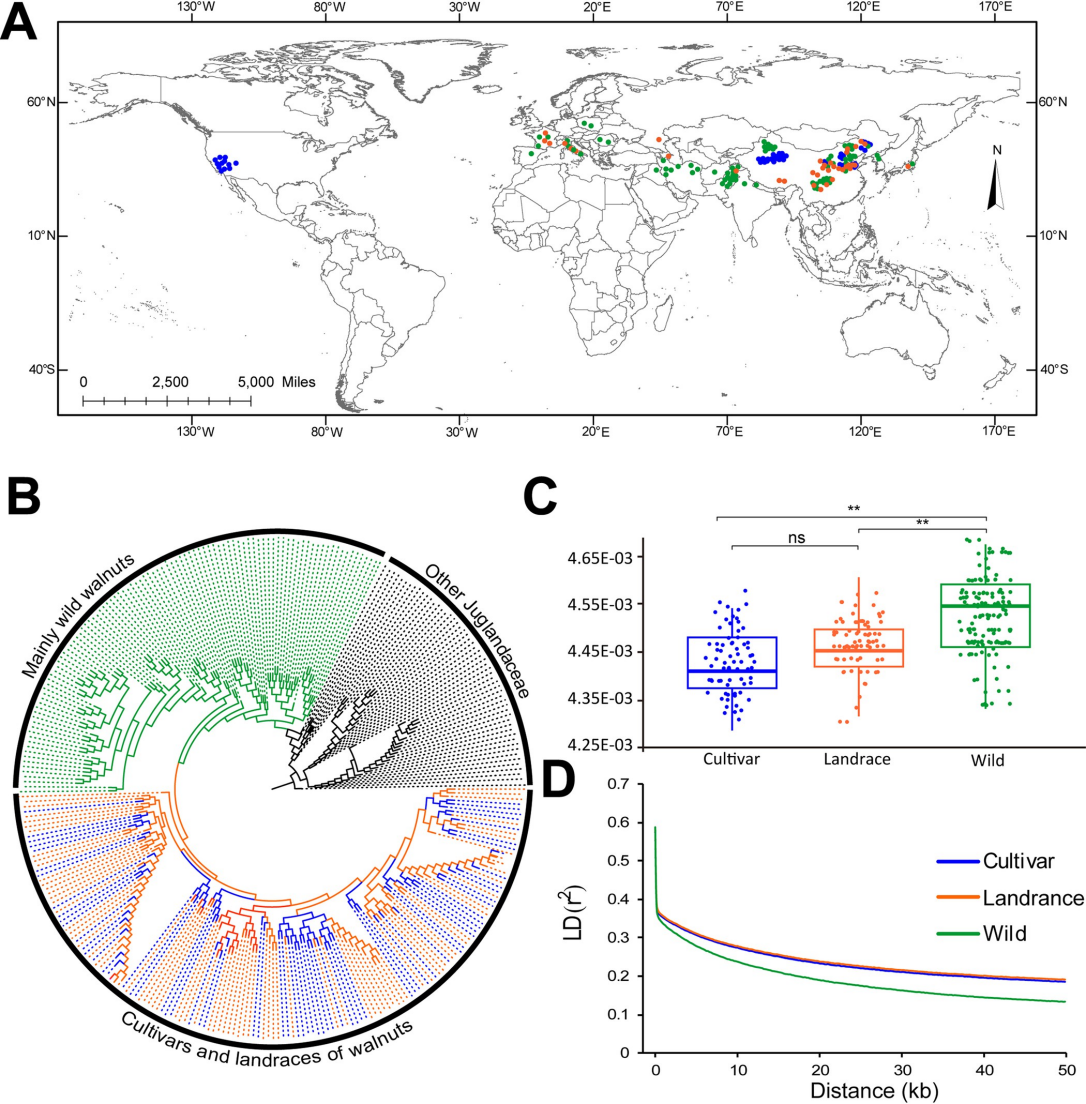
Phylogeny and population genetics of cultivated walnuts and uncultivated walnuts. (A) 309 walnut accessions distribution in different geographical regions. Green: wild; orange: landraces; blue: cultivars; and black: related *Juglans* species. The basemap and distribution of all sampling points were plotted in ArcGIS v10.2 (ESRI Inc., Redlands, California, USA). The world map was downloaded from the Natural Earth (https://www.naturalearthdata.com/downloads/10m-cultural-vectors). The grey line indicates the country region border shape in the map. (B) A neighbor-joining tree of 309 walnut accessions (C) Summary of nucleotide diversity (π) across wild walnuts, landraces, and cultivars. (D) Decay of linkage disequilibrium (LD) in different walnut groups.

The walnut populations showed relatively short linkage disequilibrium (LD) distances and relatively rapid LD decay (Fig 1D). The LD levels of the population were relatively low, with an average *r*^2^ value of 0.225 (S2 Table). The average distance over which LD decayed to ~50% of its maximum value was much shorter (3.8 kb) in wild walnuts than in landrace walnuts (7.6 kb) and cultivated walnuts (7.4 kb) (S2 Table). Cultivars and landraces were similar in terms of rates of LD decay (Fig 1D).

The results that support the distinctiveness of the wild samples of *J*. *regia* compared to the cultivated and landrace samples include differences in the π value and LD distance, but similar levels between cultivated and landrace walnuts (Fig 1C and 1D; Table 1). This provided evidence of diversity loss associated with domestication but a relatively weak additional loss of diversity due to cultivar selection.

### Phylogenetic relationships and population structure of all walnut accessions

To explore the relationships between various walnut accessions, we constructed a phylogenetic tree of all 309 accessions based on ~7.8 M SNPs. The tree revealed the presence of three distinct groups for all 309 accessions, namely, other Juglandaceae species (wild relatives of *J*. *regia*, including 55 samples), mainly wild *J*. *regia* (98 samples), and cultivar and landrace walnuts (156 samples) (Fig 1B).

**Fig 2:**
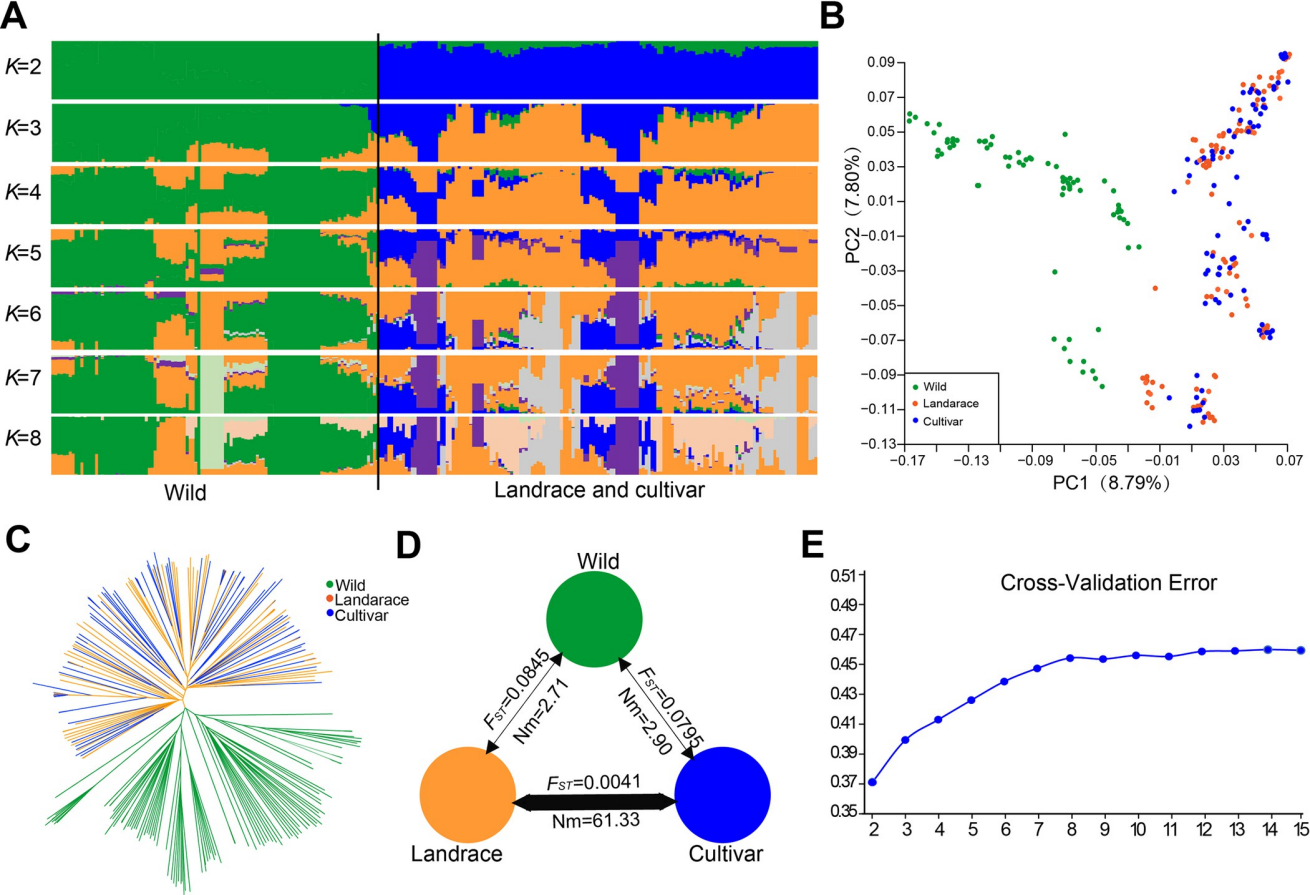
Population analysis of 254 Persian walnuts (*Juglans regia*). (A) Population structure analysis of ancestry components for 254 Persian walnuts (*Juglans regia*). Each color corresponds to a single population as noted. Each accession is represented by a vertical bar. The Y-axis refers to the proportion of the genetic background, and the height of each line with different colors represents the probability of an accession belonging to a different genetic background. (B) PCA of 254 Persian walnuts (*Juglans regia*). The first two principal components were accounting for 16.59% of variations. (C) A neighbor-joining tree of 254 walnut accessions. (D) D statistics for comparisons among Persian walnut groups. The black arrow line indicates the Z-score between the two groups. The thickness of the line indicates the value of Nm. (E) Estimated Delta K of possible clusters (k) from 2 to 15.

To further reveal the relationships of 254 *J*. *regia* accessions, we performed phylogenetic analysis, principal component analysis (PCA), and population structure analysis (Fig 2). As a result, cultivars and landraces (local varieties) were identified as belonging to a common gene pool (Fig 2A and 2C). We speculate that these individuals represent the mixing of germplasm pools as a consequence of human-mediated dispersal over large distances, possibly as part of commercial activities. The modern cultivars that we sampled are representatives of breeding programs that have drawn on a diverse base of landraces preselected for traits by local communities in geographically distinct regions. The gene flow analysis showed strong gene flow (Nm = 61.33) between landraces and cultivars by the BABA-ABBA test and Nm calculation (Fig 2D). The gene flow of wild comparing to landrace was 2.71, while gene flow was 2.90 between wilds and cultivars (Fig 2D). We also detected the same gene flow patterns by the TreeMix analysis (S2 Fig).

### Origin and differentiation of Persian walnuts

**Fig 3:**
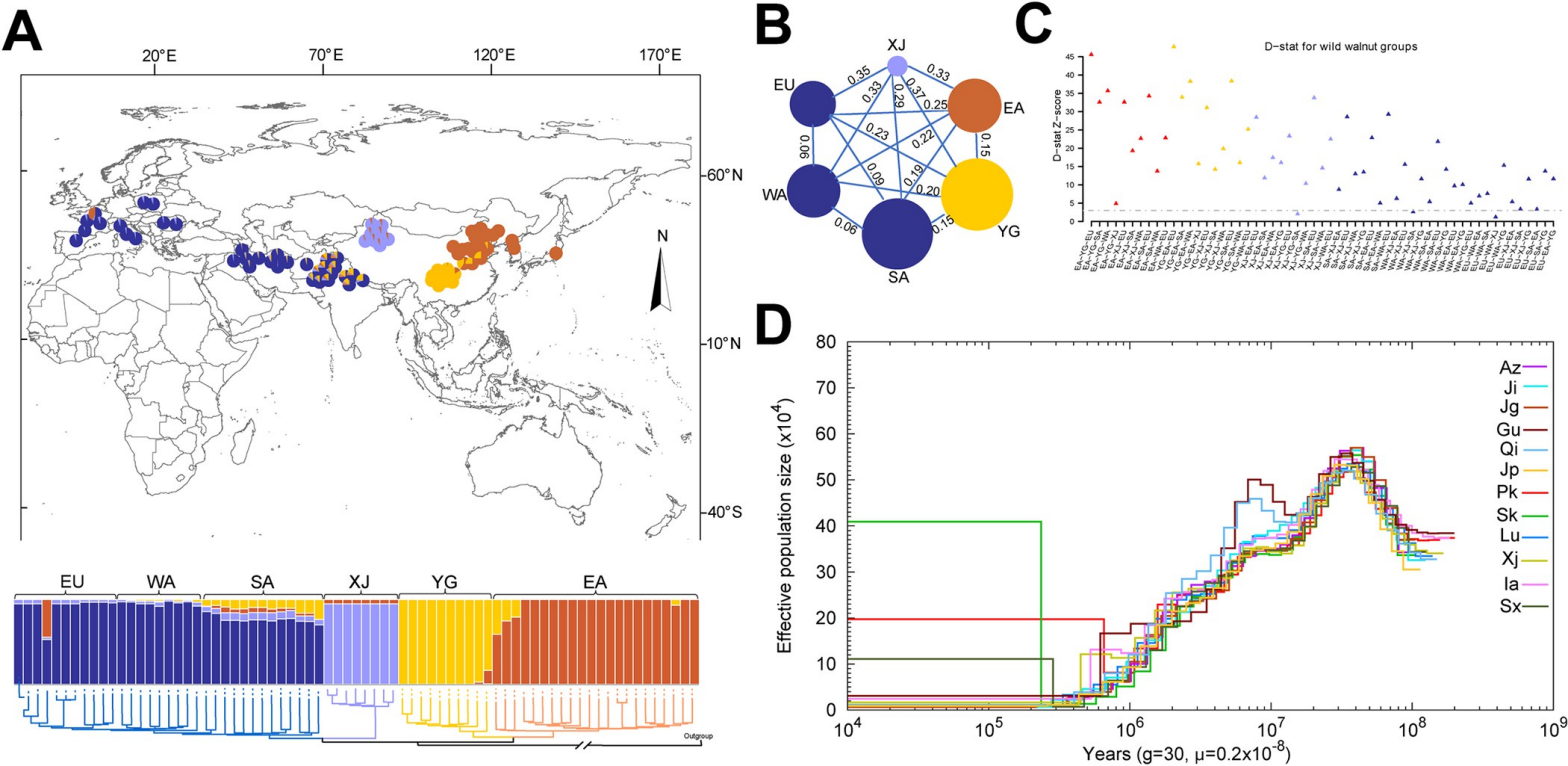
Phylogeny, population structure, diversity, and dynamic history of wild walnuts. (A) Geographic distribution, population structure, and phylogenetic tree analysis of wild Persian walnut accessions plus outgroups. Each of the six regional walnut populations are indicated by different colors. EA: Eastern Asia; YG: Yunnan-Kweichow Plateau; XJ: Xinjiang province; SA: southern Asia; WA: western Asia; EU: Europe. The basemap and distribution of all sampling points were plotted in ArcGIS v10.2 (ESRI Inc., Redlands, California, USA). The world map was downloaded from the Natural Earth (https://www.naturalearthdata.com/downloads/10m-cultural-vectors). The grey line indicates the country region border shape in the map. (B) Summary of nucleotide diversity (θπ) and population divergence (*F*_ST_) across groups. The size of each circle represents the nucleotide diversity (θπ)for the group, and values on the line between pairs indicate the population divergence (*F*_ST_). (C) Comparisons among wild Persian walnut groups (EA, YG, XJ, SA, WA, and EU) based on D statistics. The grey dotted line indicates where a Z-score is more than 3. (D) Pairwise sequentially Markovian coalescent (PSMC) estimates of the changes in the effective population size over time for 12 individuals of Persian walnut. Each line represents one individual. 10*6 = 1.0 Ma; Persian walnut Ne curves converged 1.0 million years ago, probably reflecting the time when all sources of *J*. *regia* last shared a common ancestor. “Az”-Landrace from Azerbaijan; Ji-Cultivar Jiling province, China; Jg-cultivar from Beijing, China; Gu-wild walnut from Guizhou province, China; Qi-wild walnut from Qinling mountains, China; Jp-landrace from Japan; Pk-wild walnut from Pakistan; Sk-landrace from South Korea; Lu-cultivar from Shandong province, China; Xj-cultivar from Xinjiang province, China; Ia-wild walnut from Iran; Sx-wild walnut from Shaanxi province, China.

To reveal the origin and differentiation of Persian walnuts, we focused on only wild genotypes (N = 98) and excluded all cultivated and landrace walnut accessions. The filtered SNP dataset of wild walnut accessions (8,387,336 SNPs) was subjected to phylogenetic analysis, population structure analysis (Fig 3A), and PCA (S3 Fig). The Δ*K* values revealed an optimal *K* of four sub-populations (S4 Fig), which was similar to the pattern observed in our PCA score plot (S3 Fig). The first sub-populations consisted of East Asian (EA) walnuts representing a group of samples from central and northern China. The second group contained samples from the Yunnan-Kweichow Plateau, southwestern China (YG: Yunnan and Guizhou provinces). The third group included Xinjiang walnuts represented by eight wild trees from Gongliu County in Xinjiang Province, western China (XJ: Gongliu County, Xinjiang Province), which is an apple diversity centre [22], and the fourth group consisted of South Asian (SA: Pakistan, India, and Nepal), western Asian (WA), and European (EU) walnuts. Among wild walnuts, the second group (YG) had higher nucleotide diversity (6.88 × 10^−3^) than the first group (EA, 4.01 × 10^− 3^) and the fourth group (SA, 5.76 × 10^−3^; WA: Iran, Afghanistan, and Iraq, 4.91 × 10^− 3^; EU, 4.39 × 10^− 3^). The lowest nucleotide diversity was observed among the wild walnuts in the third group (XJ, 2.48 × 10^− 3^) (Fig 3B and Table 2). Our data indicated that southwestern China and southern Asia near the Qinghai-Tibet Plateau and Himalayas represent core areas of genetic diversity of Persian walnut.

**Table 2:** Genetic diversity and *Tajima’s* D in wild Persian walnuts.

Walnut accessions- groups	Number of samples	SNP number	SNP/kb	θw	θπ	*Tajima’s* D
Eastern Asian (EA)	39	10,912,185	19.01	4.62×10^3^	4.01×10^3^	-0.56
Yungui (YG)	14	13,559,894	23.62	6.13×10^3^	6.88×10^3^	1.54
Xinjiang (XJ)	8	3,291,229	5.73	1.74×10^3^	2.48×10^3^	0.47
SA (Southern Asian)	17	13,282,721	23.14	6.10×10^3^	5.76×10^3^	-0.28
WA (Western Asian)	9	8,991,135	15.66	4.58×10^3^	4.91×10^3^	0.26
EU (Europe)	11	8,223,014	14.33	3.95×10^3^	4.39×10^3^	0.42

Note: EA: Eastern Asian; YG: Yunnan-Kweichow Plateau; XJ: Xinjiang province; SA: southern Asian; WA: western Asia; EU: Europe. SNP: single nucleotide polymorphism was comparison with ’Chandler’; SNP/kb: The numbers of SNP per kilobasepairs; θw-the Watterson’s estimator; θπ-the average pairwise divergence within a population

The results of the pairwise sequentially Markovian coalescent (PSMC) model indicated that the Persian walnut demes shared strongly similar trajectories of effective population size (*N*_*e*_) before 1.0 Mya (million years ago), but the shapes of the N_e_ curves diverged c. 0.4–0.5 Mya (Fig 3D). Most lineages’ N_e_ started to increase from *c*. 40 to *c*. 50 Mya and decreased quickly beginning at *c*. 20 Mya, while walnuts from Qinling mountains (Qi) and Guizhou Province (Gu) had different *N*_*e*_ curves during *c*. 10 Mya to *c*. 5 Mya (Fig 3D). During the period from 20.0 to 1.0 Mya (early Pleistocene), the similarity of the N_e_ dynamics of all Persian walnuts was pronounced; most walnuts reached their largest population size (N_e_~1.5–2.0⊆10^4^) *c*. 10.0 Mya. During the Quaternary glacial (1–2 Mya) in Asia, all of the Persian walnut populations that we studied showed expected glacially induced decline (Fig 3D).

High levels of gene introgression were detected between EA and YG, between WA and SA, and between WA and EU (Fig 3C; S3 Table), a result supported by the STRUCTURE results (Figs 3A and S3). The XJ population showed the lowest gene flow with other walnut populations (Fig 3C; S3 Table). We detected the highest genetic differentiation (*F*_ST_ = 0.33) value between XJ samples and samples from other populations; the lowest *F*_ST_ values were found between WA and SA and between WA and EU (Fig 3B). The PCA results showed that the XJ population was surprisingly distinct from all other populations; the distinctiveness of the XJ samples was also evident in the STRUCTURE results at *K* = 3 to *K* = 6 (S4 Fig). The sample number of wild trees from XJ was small because wild trees are difficult to access there; conclusions based on this small sample must be considered tentative. The population structure analysis showed that the samples from South Asia (SA) exhibited considerable admixture from all other sources (east and west) (Figs 3A and S4). It is possible that a more comprehensive sampling of remote locations will reveal the presence of genetic diversity not shared by other sources. The European and western Asian populations displayed low levels of gene introgression from SA and YG (Figs 3A and S4). Walnut diversity in Eurasia is globally governed by the separation between populations in eastern and southwestern China (EA and YG) and all other populations.

### Genome-wide selection and GWAS of genes during domestication

**Fig 4:**
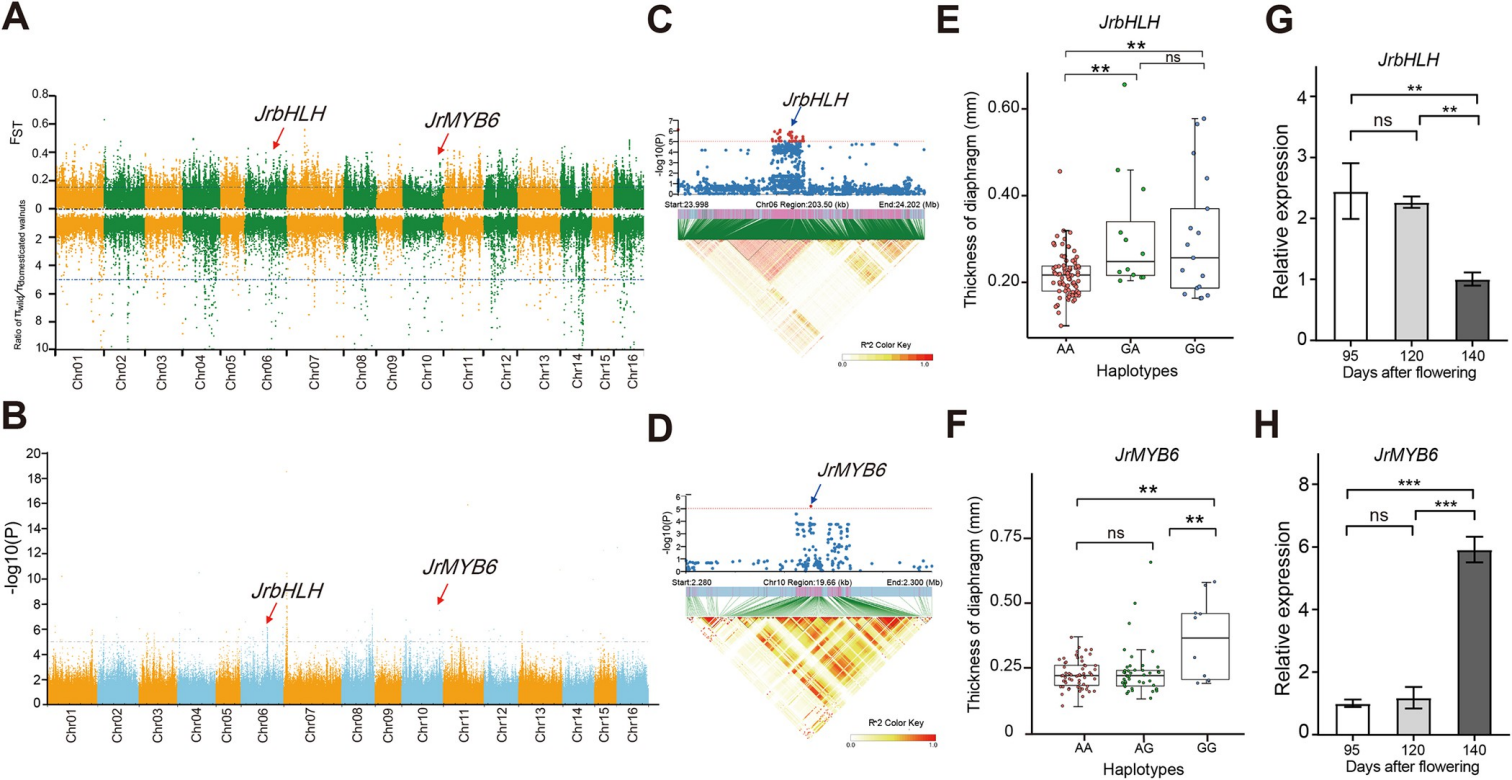
Selective sweeps and GWAS of diaphragm thickness. (A) Distribution of the *F*_ST_ and nucleotide diversity (ROD) for the wild group and cultivar+landrace group (π_wild_/π_cultivar+landrace_; domesticated) in windows along chromosomes. Horizontal dotted lines represent the cutoff fulfilling the requirement for regions under selection. The dashed line indicates the *F*_ST_ = 0.15. The red arrow indicates the selected loci in *JrbHLH* and *JrMYB6*. (B) Manhattan plot for GWAS on the thickness of the diaphragm in the full population. The dashed line indicates the threshold −log(P) = 5. The red arrow indicates the SNP in *JrbHLH* and *JrMYB6*. Manhattan plot (top) and linkage disequilibrium heat map (bottom) for *JrbHLH* (C) and *JrMYB6* (D). Box plots show the thickness of the diaphragm in three haplotypes (Hap.) of *JrbHLH* (E) and *JrMYB6* (F). Relative expression levels of TF *JrbHLH* (G) and *JrMYB6* (H) in three development stages of the walnut diaphragm. **P < 0.01, *** *P* < 0.001, ns = not significant, Student’s test-test.

As in most fruit tree species, an important step in the domestication of wild walnut into a nut crop must have involved the (partial) adaptation to cultivation and local environments through human selection. Genome-wide selection analysis indicated that approximately 3% (16.4 Mb) of the walnut genome containing 866 genes were shaped by selection based on a comparison of wild versus cultivar+landrace groups (Fig 4A; S4 Table). Among the genes under selection, *JrbHLH* was located 119 bp downstream of the associated SNP (Loci: 24,101,955) on chromosome 6, and *JrMYB6* was located 951 bp upstream of the associated SNP (Loci: 2,290,478) on chromosome 10 based on GWAS for the thickness of the nut diaphragm (Fig 4B, 4C and 4D). These two SNPs could successfully distinguish the A and G alleles in the association panel with the thickness of the nut diaphragm (Fig 4E and 4F). The nut diaphragm thickness of the lines with the G allele were significantly higher than in those with the A allele (*P* < 0.01). Sequence comparison analysis indicated that the *JrbHLH* was homologs to that of *Arabidopsis thaliana*, *Glycine max*, *Populus trichocarpa*, and *Malus domestica* (S5 Fig). While *JrMYB6* of *J*. *regia* was homologs to that of *Zea mays*, *Arabidopsis thaliana*, *Solanum lycopersicum*, *Malus domestica*, *Oryza sativa*, *Glycine max*, and *Populus trichocarpa* (S6 Fig). The Expression analysis further denoted that *JrbHLH* was expressed decreased during walnut diaphragm development (July 27, August 18, and September 6), while *JrMYB6* was expressed increased during three walnut diaphragm developmental stages (Fig 4G and 4H). Thus, these two genes are selected and may play important roles during the development of the walnut diaphragm.

## Discussion

Here, we sequenced the genomes of 309 walnut (*Juglans*) samples collected around the world, with a focus on *J*. *regia*, the most valuable nut crop in China. The study revealed that the core areas of genetic diversity of Persian walnut germplasm were southwestern China and southern Asia near the Qinghai-Tibet Plateau and the Himalayas. A comparison of the genomic diversity of wild, landrace, and improved Persian walnuts showed evidence of loss of diversity associated with domestication but a relatively weak additional loss of diversity from cultivar selection. The resources generated in this study provide a genomic framework for future germplasm use and walnut improvement.

### Genetic diversity in Persian walnuts

When we began this research, we expected that close examination would show that breeders had reduced the genetic diversity of walnut as they advanced germplasm into the final stages of cultivar development. In general, this was not true; the genetic diversity, LD distance, and gene flow provided a relatively weak artificial selection in the evolution process, which is different from the main annual crops like rice and soybeans [18–20]. This could be explained by the outcrossing system, domestication history, and modern breeding history. First, walnut is wind pollination and heterodichogamous characteristics of the breeding system [5–6,12], perennial and long juvenile phase, which is similar to some other woody fruit trees (e.g., pear, apple, peach, and jujube) [22–25,32–36]. Second, from the point of domestication history, the divergence time between wild walnuts vs. landrace and cultivar was much later than annual crops such as rice and soybean. *J*. *regia* is considered as food used by humans back to Persia (7,000 BCE) [7,9,12,16], however, as an important annual crop, both rice and soybean ancient human domestication started from ~8,000 BCE [19–20,37–39]. Moreover, the walnut population had declined by the bottleneck scenario [40], which limited the expansion of genetic diversity. Third, intensive breeding may not be necessary where landraces already expressed desirable commercial traits to a considerable degree. Modern cultivars have a short pedigree and are generally developed by several breeding programs around the world from a geographically and genetically diverse group of landraces. In addition, most modern walnut cultivars have relatively short pedigrees because breeding programs have managed only a few generations of selection in the past 70 years or so, as walnut is perennial woody and has a long generation time. Importantly, the phenomenon of weak selection reminds us that it should be cautious during artificial selection breeding as wild material can also be feral accessions.

### Phylogenetic relationships and gene introgressions of walnuts

Within *Juglans*, the phylogenetic tree showed that the *J*. *regia* samples (section *Dioscaryon*) were monophyletic (Fig 1B). Distinct and parallel clades were seen for the sect. *Cardiocaryon*, sect. *Trachycaryon*, and sect. *Rhysocaryon* based on our walnut samples (Fig 1B). The systematic position of Persian walnut in the genus *Juglans* has been well resolved based on morphology and molecular evidence, including sequence data from the internal transcribed spacer (ITS), five chloroplast DNA spacer sequences, a hypervariable *matK*, restriction fragment length polymorphisms (RFLPs), and whole chloroplast genomes [4–5,15,41–44]. The origin of sect. *Dioscaryon* is a topic of active research; however, it may be that *J*. *regia* and other members of *Dioscaryon* were derived from the hybridization of ancestors that became what is now sect. *Rhysocaryon* and sect. *Cardiocaryon*, a conclusion based on nuclear SNPs [11,45]. Differences in the evolutionary rate, inherited background, and complex speciation history of *Juglans* may explain why the phylogeny of Persian walnut differs depending on whether it is based on nuclear SNPs or chloroplast and mitochondrial sequences [46–48]. The tree that we generated based on SNPs was consistent with previous conclusions based on nuclear data and chloroplast genomes [43–48]. Therefore, our whole-genome resequencing data offer a valuable resource for examining the taxonomy of *J*. *regia* within the genus *Juglans*.

We observed that the landraces and cultivars were mixed and showed signs of introgression, certainly because of the use of landraces for cultivar breeding, as mentioned above, but probably also because seeds or scion wood of cultivars was propagated by villagers, which permitted gene exchange through pollen [2]. The gene flow analysis showed strong gene flow between landraces and cultivars by the BABA-ABBA test, Nm calculation, and TreeMix analysis (Figs 2D and S2). Based on studies of other tree species, local gene flow between wild and cultivated individuals is an important evolutionary factor [49–52]. Walnut landraces propagated by local communities as seedlings represent a valuable resource for breeders as a secondary germplasm pool [8].

### The demographic history and past ecological distribution shifts of Persian walnut

Our results are supported by previous genetic and ecological data showing that Persian walnut survived the LGM in glacial refugia spread across a wide geographic area from 30°N to 45°N latitude in the Balkans, western Europe, Xinjiang Province, northeastern China, central China, and southwestern China [7,10,12]. During the Pleistocene, the differentiation of populations may have been influenced by repeated extinction and colonization during the Quaternary climate oscillations [12,53].

The presence of walnut habitat in the mountains of southern Asia was supported by a recent archaeological study of several new sites in northeastern India containing carbonized walnut samples ascribed to an earlier date (~4500 B.P.) than previously reported (~4000–3500 B.P.) [54]. The wide distribution of common walnuts in broadly similar but fragmented environments across Asia during the Late Miocene and Pleistocene climate changes [7,12] may have resulted in regionally adapted populations with an effective population size continually constrained by climate fluctuations and the long generation time of this perennial nut crop [22,54–55]. The formation of the Qinghai-Tibet Plateau and climate changes were the important drivers of the long-term population dynamics of Persian walnut [56–57].

Persian walnut is considered a relict species of the Tertiary Period [7,10,12] native to the mountain ranges of Asia [11,27,47]. Here, we found that the Qinghai-Tibet Plateau in southwestern China (including southern Asia and southwestern China) may be the core region of genetic diversity (Fig 3B; Tables 1 and S2). Taking these results together, it is inferred that Persian walnut found worldwide differentiated from an ancestral Qinghai-Tibet Plateau population. This region is where the only other taxon in *Dioscaryon* (*J*. *sigillata*) is found [58]. The current distribution of walnut is the result of expansion/contraction from multiple refugia during the uplift of the Himalayan Mountains, climate changes, ecological adaptation [53,59–60], and later human exploitation [61–63].

### Genes under selection

To the best of our knowledge, the diaphragm is a typical domestication trait that protects the nuts during natural environmental selection, thereby maintaining the continuity of the species. In addition, the diaphragm affects the ease of kernel extraction, and a thin diaphragm shows the great commercial value and thus is attractive to breeders. In the study, selection analysis and GWAS revealed that *JrbHLH* and *JrMYB6* were associated with the thickness of the diaphragm in walnut. The homologs of *JrbHLH* in poplar was related to cell wall development [64–65]. The homologs of *JrMYB6* in *Malus domestica* is involved in relative lignin metabolism [66–67]. It is known that lignin is a principal structural component of cell walls in higher terrestrial plants [68]. Thus, *JrbHLH* and *JrbMYB6* may regulate cell wall formation and lignin biosynthesis during xylem development in walnut. Additionally, expression analysis indicated that *JrMYB6* was upregulated in walnut diaphragm development, but *JrbHLH* was downregulated during walnut diaphragm development. Collectively, *JrbHLH* and *JrMYB6* might differentially express to participate in the xylem development to affect the thickness of the diaphragm in walnut. Thus, these two TFs provide potential targets for improving Persian walnut nut quality by molecular marker-assisted selection or genetic manipulations.

## Materials and methods

### Sample collection and preparation

Wild samples were collected from apparently healthy mature adults growing in mountain forests, near villages, or along forest roads but not in orchards or on farmed land. The sampled trees were separated by at least 50 m. Landrace samples were collected from different local communities, while the cultivar samples were collected from orchards containing the named cultivars. A total of 55 Persian walnut relatives, including 23 black walnuts (*J*. *nigra*), 20 butternuts (8 *J*. *cathayensis*, 8 *J*. *mandshurica*, and 4 *J*. *cinerea*), 11 hybrids (*J*. *hopeiensis*), and *Carya illinoinensis* (S1 Table), were obtained from improvement programs from many countries and were presumably selected to produce high-quality nuts in a wide range of production environments. The distribution of all sampling points was plotted with ArcGIS v10.2 (ESRI Inc., Redlands, California, USA). DNA extraction was performed using the improved CTAB method of Zhao and Woeste (2011) [69]. From 2018 to 2019, we measured the thickness of the diaphragm in Persian walnut accessions grown in Tai’an, Shandong Province, China (S1 Table). Three nuts from each accession in each replication were used for the measurements of diaphragm thickness.

### DNA sequencing and detection of variations

In total, 5169 Gb of raw data was obtained using paired-end 150 bp reads. Libraries were processed with the Illumina Cluster Generation Station following the manufacturer’s recommendations and sequenced on a HiSeq 4000 instrument. The CASAVA 1.7.0 version of the Illumina pipeline was used to process raw data [70].

To obtain clean reads, we filtered the reads with three features: 1) reads with adapters, 2) reads with low quality (<10) base percentage greater than 20% compared to the whole genome, and 3) reads with N percentages greater than 5%. After filtering, we mapped the clean reads to the reference genome using BWA V0.7.17 software with default parameters [71]. Using the *J*. *regia* ’Chandler’ (updated version 2.0) chromosome-level genome as a reference [26], clean data were mapped to detect SNPs and InDels for all 309 genotypes. Based on high-quality alignment (Map Quality>20), we used Genome Analysis ToolKit V4.1.1.0 model *haplotypecaller* to identify SNPs [72]. Finally, to ensure data quality, we kept the SNPs that met the following criteria: 1) biallelic SNPs, 2) SNPs with a quality score >30, 3) SNPs with a missing rate (MR) <0.25, and 4) SNPs with a minor allele frequency (MAF) >0.05.

### Genetic diversity, population structure, and LD analysis

To compare the diversity of *J*. *regia* groups around the world, we wrote an in-house Perl script (https://github.com/xujiabao127/Walnut/) to calculate the diversity parameters, including the SNP number, the average pairwise divergence within a population (θ_π_), Watterson’s estimator (θ_w_) and *Tajima’s D*. Along the genome, a sliding window of 10 kb with a 5 kb step was used to estimate the θ_π_, θ_w_, and *Tajima’s D* values. The population differentiation parameter *F*_ST_ and reduction of diversity (ROD) were computed in the same windows based on a pairwise SNP sequence [73]. PHYLIP software (version 3.696) was used to produce a cladogram based on the genetic distance matrix using the p-distance formula [74]. The algorithm we chose was the neighbour-joining (NJ) method. We set *C*. *illinoinensis* as the outgroup. Figtree was used to graph the phylogenetic tree. We performed PCA using PLINK software (version 1.90) with default settings [75]. The top four eigenvectors were selected for the graph shown by EIGENSOFT software [76]. The population structure of 98 wild Persian walnut samples and 254 wild, cultivar, and landrace samples was investigated using ADMIXTURE software (version 1.3.0) [77] with a marginal likelihood model. We ran 10,000 iterations, and the number of clusters (*K*) was set from 2 to 10, representing the assumed groups of the simulated ancestral population. The best *K* was inferred based on the delta *K* method using the Structure Harvester program [78]. LD was calculated using PLINK software (version 1.07) [75] with the following settings:—file—r2—ld-window 99999—ld-window-kb 200—out. Then, values for the r2 statistics were obtained. LD decay was calculated based on r2 between two SNPs and the distance between the two SNPs.

### Population history

We used the PSMC model to infer the population size history of different groups. First, heterozygous sites were generated by SAMtools version 1.6 [79]. The parameters were *“samtools mpileup -C 50 -Q 20 -u -v”* and *“vcfutils*. *pl vcf2fq -d 4 -D 100”*. Second, the diploid consensus sequence was obtained by fq2psmcfa as an input of the PSMC, and the parameter was “fq2psmcfa -q20*”*. Third, *PSMC V0*.*6*.*5* was run with the parameter “psmc -N25 -t15 -r5 -p 4+25*2+4+6”. To analyze the results, we used 30 years as the generation time, and the mutation rate was set as 2.09E-8 substitutions per site per generation [20].

### Gene flow analysis between wild, landrace, and cultivar walnuts

To understand gene flow, we calculated the D-statistics of four populations with ADMIXtools version 4.1 software [77]. The SNPs in vcf format were converted to EIGENSOFT format via VCFtools version 0.1.13 and CONVERTF provided by ADMIXtools [77]. The D-statistics were computed by qpDstat. Sample s99 (*C*. *illinoinensis*) was set as the outgroup (X) for all four population tests. For any four populations (W, X, Y, and Z), the gene flow occurred either between W and Y or between X and Z when the Z score was more than 0. Conversely, gene flow occurred between either W and Z or X and Y when the Z score was smaller than 0. A gene flow signal was considered significant when the Z score was more than 3 or less than -3. To study gene flow among the wild samples, landraces, and cultivars, we calculated two parameters: the D-statistic and Nm (estimate of gene flow). The D-statistics were generated by ADMIXtools software based on the fixation index (*F*_ST_). Nm was calculated according to Wright’s (1931) formula Nm = (1- *F*_ST_)/(4*F_ST_) [80]. TreeMix22 version 1.12 was used to model the gene flow between the outgroups, wild walnuts, landrace walnuts, and cultivar walnuts [81].

### Detection of selective sweeps

To identify evidence of selection in Persian walnut genomes associated with domestication and improvement, we screened for genomic regions with a sharp ROD between wild and domesticated walnut (cultivars + landraces) groups (π_wild_/π_cultivar+landrace_; domesticated). Genomic regions under selection often showed a decrease in genetic diversity. We identified genomic regions selected by domestication by comparing the *F*_ST_ and ROD for the pool of wild trees compared to the cultivar+landrace pool using a 100-kb sliding window with a step size of 10 kb using VCFtools software (v0.1.13) [73,79,81]. The candidate selected regions associated with human cultivation were identified with the following criteria: 1) *F*_ST_>0.15 (the genetic differentiation between groups is large), 2) the top 10% of *F*_ST_ values, and 3) ROD>0.2. In the domestication-related genomic regions, we selected genes meeting these three criteria as candidate domestication genes [73,78].

### GWAS and identification of the candidate genes

The SNPs with an MAF<0.05 and a missing rate<0.25 were used for the GWAS in SHAPEIT (4.0) [82–83]. To obtain a high confidence value for each trait, we calculated average values among different years. Then, we deleted the sample with a trait value greater than mean+2 standard deviations (SDs) or less than the mean-2 SDs. To estimate the kinship among samples, we used the software tassel (V5.0) to calculate the kinship matrix [84]. We used admixture (v 1.3.0) to calculate the population structure and Q matrix for the GWAS [77]. Based on the SNPs, trait values, kinship matrix, and Q matrix, we performed the GWAS analysis by the FarmCPU model in GAPIT (V3.0) software [85]. According to the associated loci determined by the GWAS, SNP types and locations were identified using the reference genome [26]. Haploview v4.2 and LDBlockShow v1.40 software were used to construct and visualize haplotype maps [86–87].

### Gene expression analysis by qRT-PCR

To verify the expression patterns of two candidate transcription factors (*JrbHLH* and *JrMYB6*), we collected common walnut diaphragms from one adult tree growing at Xi’an (Northwest University campus) on 95 days after flowering (DAF), 120 DAF, and 140 DAF, respectively. Total RNA from walnut diaphragms was processed using RNA Library Prep Kit (Beverly, MA, USA). We then performed quantitative real-time PCR (qRT-PCR) reactions in a common walnut diaphragm during three development stages (July 27, August 18, and September 6) (primers see details in S6 Table). Before the PCR experiment, primer specificities and corresponding melting curves were verified. In each qRT-PCR experiment, three replicates were performed. Real-time amplification responses were performed on an Applied Biosystems (USA) 7500 quick real-time PCR system [88–89]. The relative concentration of expression of each gene was calculated using the 2-Ct method [88–89].

## Supporting information

S1 Table Summary of 309 walnut accessions sequenced and mapped against the ‘Chandler’ (*J*. *regia*) reference. (XLSX)

S2 Table Linkage disequilibrium (LD) between cultivar, landrace, and wild walnut samples. (XLSX)

S3 Table D statistics for comparisons among wild walnut populations. (XLSX)

S4 Table Genes identified as candidates for selection by *F*_ST_ ratio of wild and cultivated accessions, and their functional annotation. (XLSX)

S5 Table Identification and annotation of candidate genes associated with the thickness of the diaphragm. (XLSX)

S6 Table Primers used for quantitative real-time PCR. (XLSX)

S1 Fig Variation across the chromosomes of *Juglans regia* based on 300Kb windows. (A) sixteen chromosomes of *Juglans regia*. (B) Gene density represented as a grayscale heatmap; i.e., blacker areas had a higher gene density (max value was 56 single nucleotide polymorphisms (SNPs) per 300 kbp window). (C) Number of SNPs in 300-kb windows. SNPs among wild walnuts (green), cultivars (light blue), and landraces (red) (max value was 36 SNPs per 300 kbp window). (D) Insertions and deletions (indels) in wild walnuts (green), cultivars (light blue), and landraces (red). (E) The genomic nucleotide diversity (π) of the nuclear genomes of wild walnuts (green), cultivars (light blue), and landraces (red). (F) Heatmap of the genetic differentiation (*F*_ST_) between wild walnuts and selected walnuts (cultivars and landraces). (G) Heatmap of the coverage of selected regions between wild walnuts and selected walnut (cultivars and landraces).
(TIF)

S2 Fig Gene-flow patterns that were detected among walnut groups using TreeMix. JY = outgroup, Wild = wild walnuts, Landrace = landrace walnuts, Cultivated = cultivar walnuts.
(TIF)

S3 Fig The principal component analysis (PCA) of wild walnuts. Principal components plots for wild walnut accessions, including samples from Eastern Asian (EA, N = 39), Yunnan-Kweichow Plateau (YG, N = 14), Xinjiang Province (XJ, N = 8); southern Asia (SA, N = 17); western Asia (WA, N = 9); Europe (EU, N = 11).
(TIF)

S4 Fig Population structure (*K* = 2–6) of wild walnuts. (A) Each color corresponds to a single population as noted. Each walnut accession is represented by a vertical bar. The Y-axis refers to the proportion of the genetic background, and the height of each line with different colors represents the probability of an accession belonging to a different genetic background. (B) Delta K showed a peak at 4, suggesting four clusters as the most appropriate option, which supports the phylogenetic tree and PCA result of “four major discrete clusters of Eastern Asian (EA), Yunnan-Kweichow Plateau (YG), Xinjiang province (XJ), and southern Asian (SA)+western Asia (WA)+ Europe (EU) groups were detected”.
(TIF)

S5 Fig Alignment of bHLH proteins from *Juglans regia*, *Arabidopsis thaliana*, *Glycine max*, *Populus trichocarpa*, and *Malus domestica*. The protein information of these plants as follows: *J*. *regia* (XP_035546602.1), *A*. *thaliana* (AT1G69010.1), *G*. *max* (KRH03185), *P*. *trichocarpa* (PNT00429), and *M*. *domestica* (mRNA:MD06).
(TIF)

S6 Fig Alignment of MYB proteins from *Juglans regia*, *Zea mays*, *Arabidopsis thaliana*, *Solanum lycopersicum*, *Malus domestica*, *Oryza sativa*, *Glycine max*, and *Populus trichocarpa*. The protein information of these plants as follows: *J*. *regia* (XP_018812236.1), *Zea mays* (Zm00001eb392230,), *Arabidopsis thaliana* (AT3G47600.1), *Solanum lycopersicum* (Solyc06g069), *Malus domestica* (mRNA:MD06), *Oryza sativa* (Os12t0125000-01), *Glycine ma*x (KRH64960.1), and *Populus trichocarpa* (PNS90469).
(TIF)
