# Supplementary figures and images for: Domestication and selection footprints in Persian walnuts (Juglans regia)
Source: PLoS Genet. 2022 Dec 7;18(12):e1010513. doi: 10.1371/journal.pgen.1010513 (PMC9728896; doi:10.1371/journal.pgen.1010513)

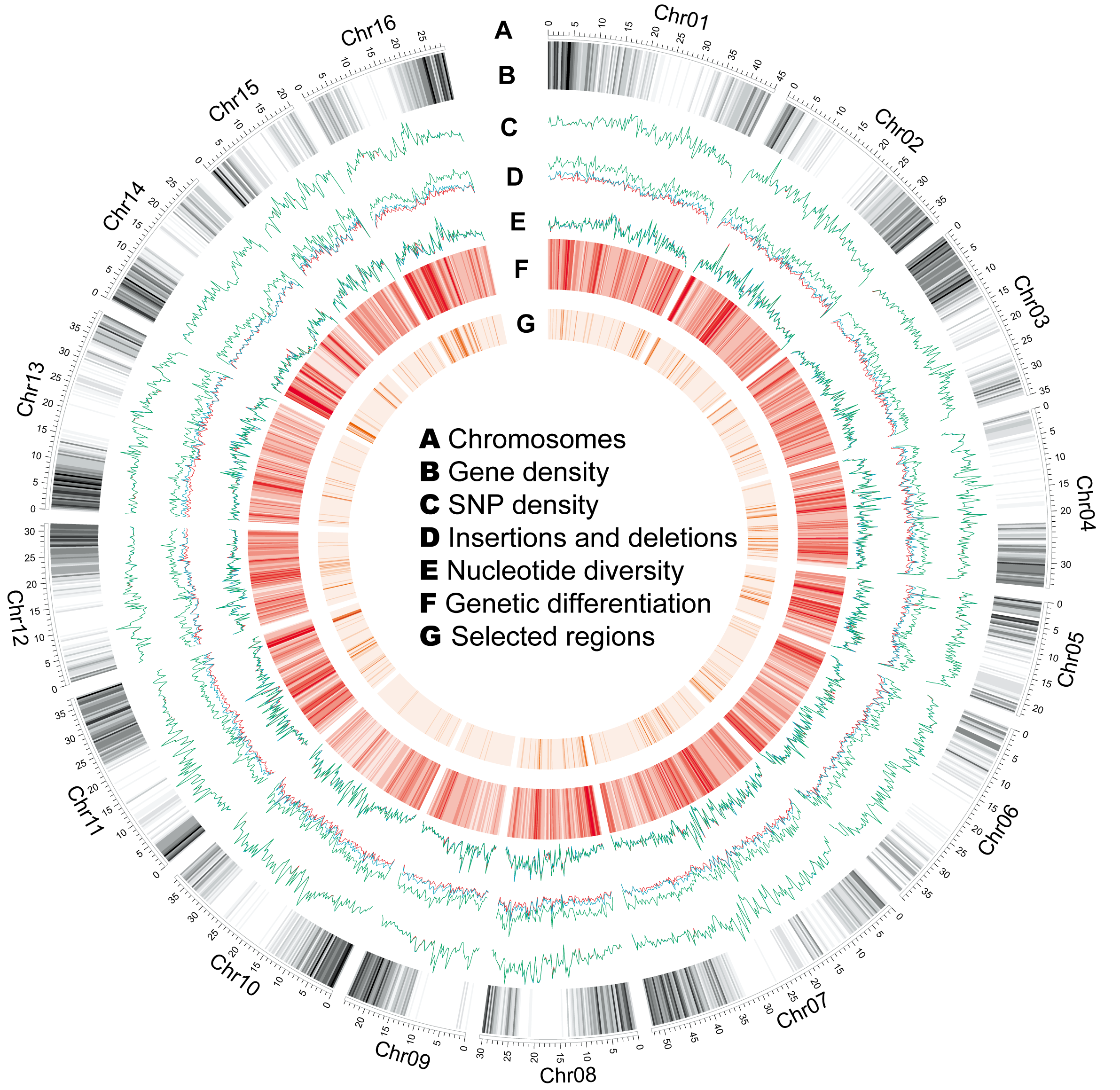

Supplement: S1 Fig — (A) sixteen chromosomes of Juglans regia. (B) Gene density represented as a grayscale heatmap; i.e., blacker areas had a higher gene density (max value was 56 single nucleotide polymorphisms (SNPs) per 300 kbp window). (C) Number of SNPs in 300-kb windows. SNPs among wild walnuts (green), cultivars (light blue), and landraces (red) (max value was 36 SNPs per 300 kbp window). (D) Insertions and deletions (indels) in wild walnuts (green), cultivars (light blue), and landraces (red). (E) The genomic nucleotide diversity (π) of the nuclear genomes of wild walnuts (green), cultivars (light blue), and landraces (red). (F) Heatmap of the genetic differentiation (FST) between wild walnuts and selected walnuts (cultivars and landraces). (G) Heatmap of the coverage of selected regions between wild walnuts and selected walnut (cultivars and landraces). (TIF) [file pgen.1010513.s007.tif]

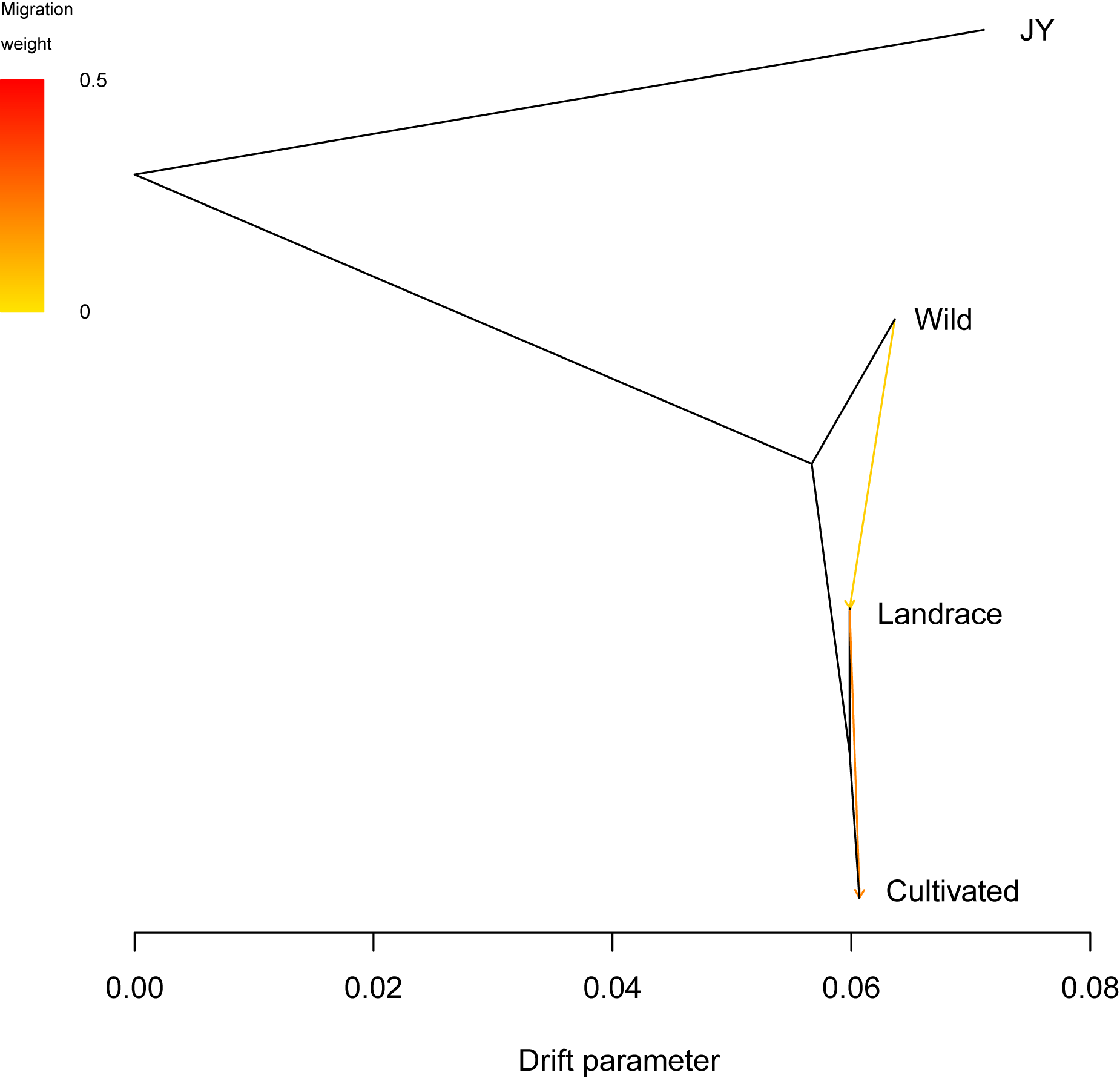

Supplement: S2 Fig — JY = outgroup, Wild = wild walnuts, Landrace = landrace walnuts, Cultivated = cultivar walnuts. (TIF) [file pgen.1010513.s008.tif]

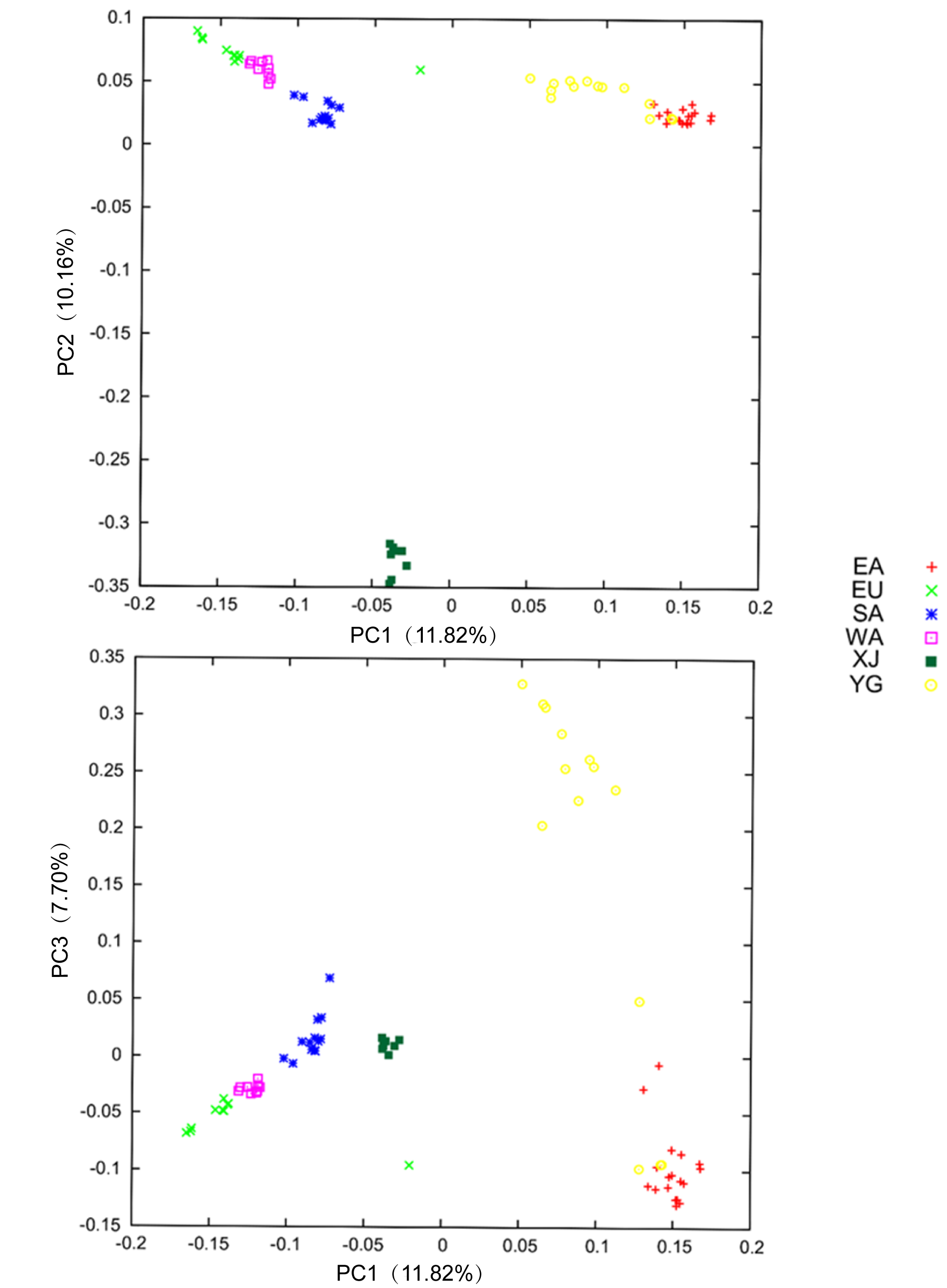

Supplement: S3 Fig — Principal components plots for wild walnut accessions, including samples from Eastern Asian (EA, N = 39), Yunnan-Kweichow Plateau (YG, N = 14), Xinjiang Province (XJ, N = 8); southern Asia (SA, N = 17); western Asia (WA, N = 9); Europe (EU, N = 11). (TIF) [file pgen.1010513.s009.tif]

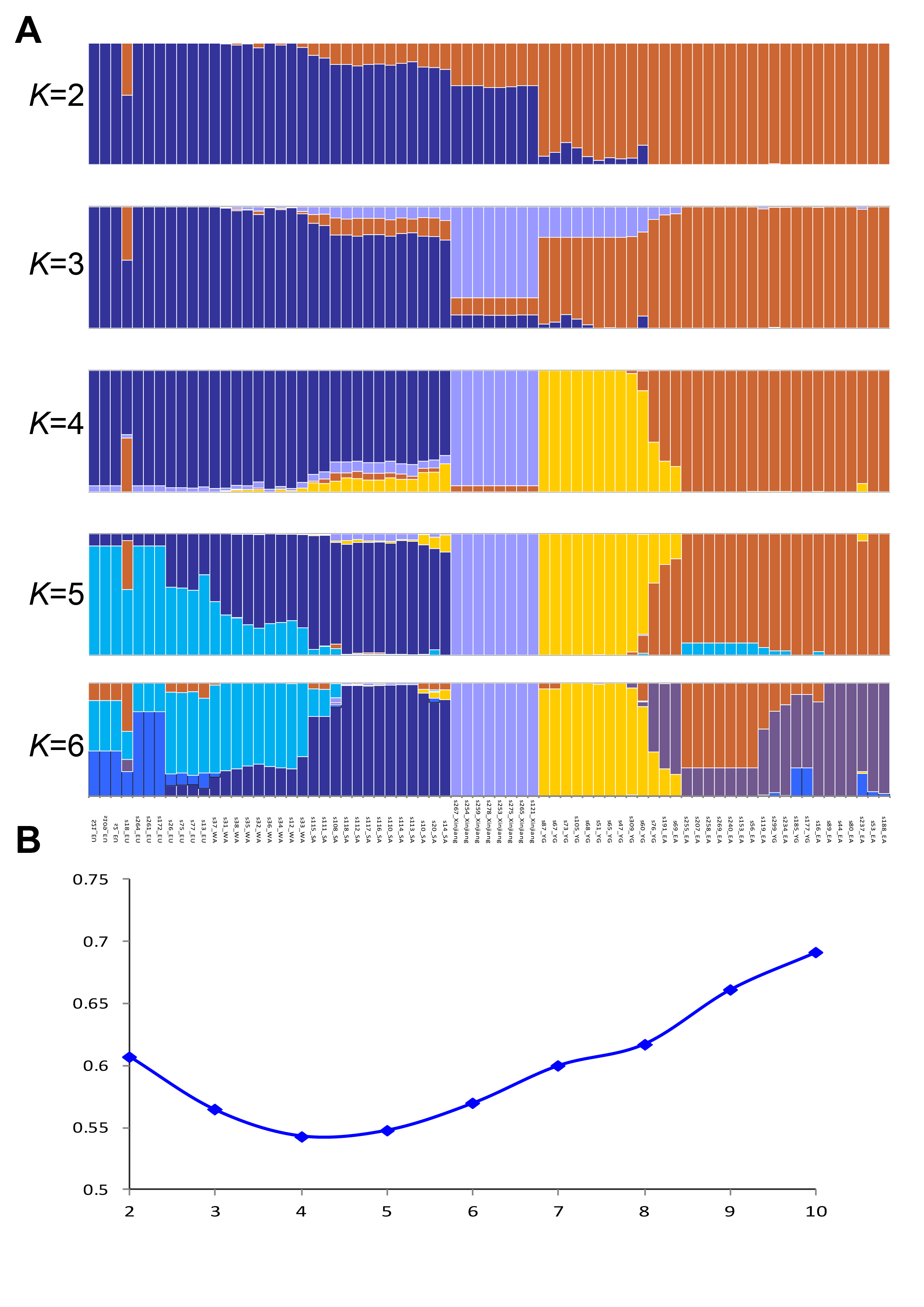

Supplement: S4 Fig — (A) Each color corresponds to a single population as noted. Each walnut accession is represented by a vertical bar. The Y-axis refers to the proportion of the genetic background, and the height of each line with different colors represents the probability of an accession belonging to a different genetic background. (B) Delta K showed a peak at 4, suggesting four clusters as the most appropriate option, which supports the phylogenetic tree and PCA result of “four major discrete clusters of Eastern Asian (EA), Yunnan-Kweichow Plateau (YG), Xinjiang province (XJ), and southern Asian (SA)+western Asia (WA)+ Europe (EU) groups were detected”. (TIF) [file pgen.1010513.s010.tif]

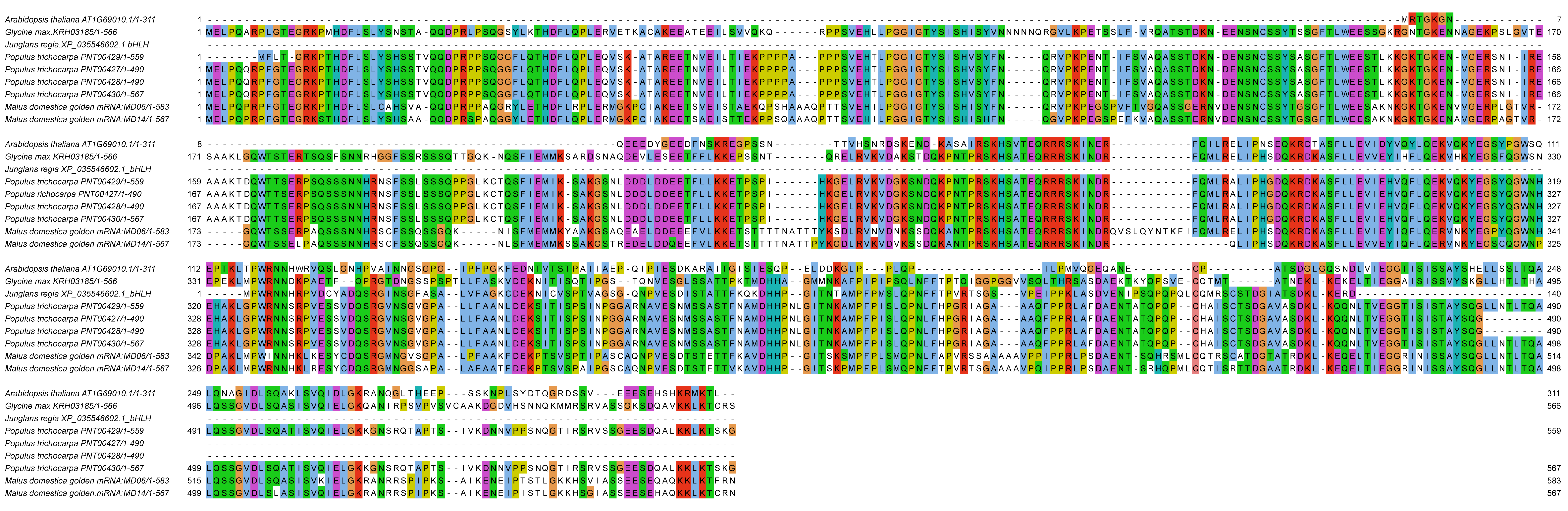

Supplement: S5 Fig — The protein information of these plants as follows: J. regia (XP_035546602.1), A. thaliana (AT1G69010.1), G. max (KRH03185), P. trichocarpa (PNT00429), and M. domestica (mRNA:MD06). (TIF) [file pgen.1010513.s011.tif]

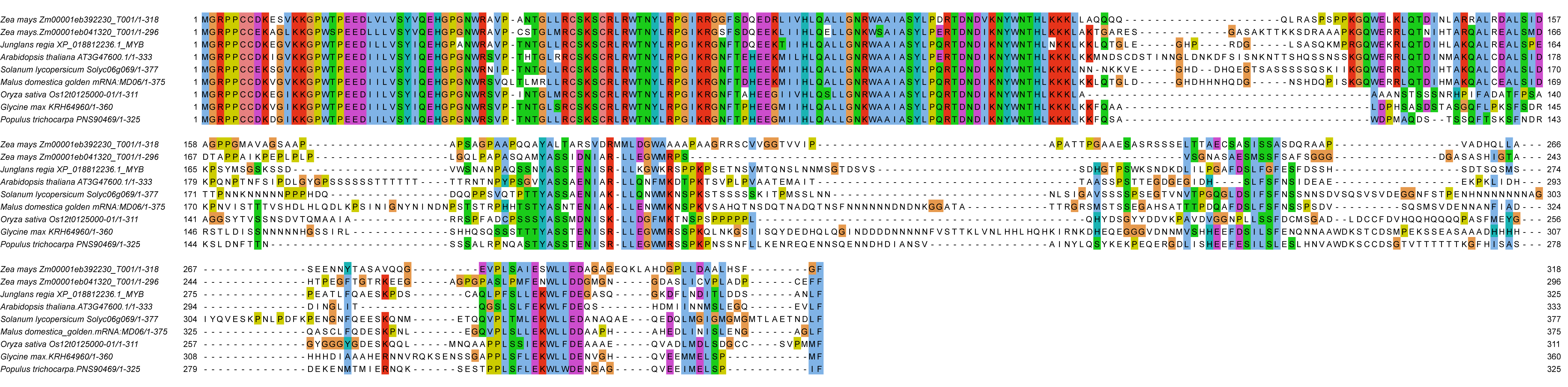

Supplement: S6 Fig — The protein information of these plants as follows: J. regia (XP_018812236.1), Zea mays (Zm00001eb392230,), Arabidopsis thaliana (AT3G47600.1), Solanum lycopersicum (Solyc06g069), Malus domestica (mRNA:MD06), Oryza sativa (Os12t0125000-01), Glycine max (KRH64960.1), and Populus trichocarpa (PNS90469). (TIF) [file pgen.1010513.s012.tif]
